# Artificial intelligence for quantum computing

**DOI:** 10.1038/s41467-025-65836-3

**Published:** 2025-12-02

**Authors:** Yuri Alexeev, Marwa H. Farag, Taylor L. Patti, Mark E. Wolf, Natalia Ares, Alán Aspuru-Guzik, Simon C. Benjamin, Zhenyu Cai, Shuxiang Cao, Christopher Chamberland, Zohim Chandani, Federico Fedele, Ikko Hamamura, Nicholas Harrigan, Jin-Sung Kim, Elica Kyoseva, Justin G. Lietz, Tom Lubowe, Alexander McCaskey, Roger G. Melko, Kouhei Nakaji, Alberto Peruzzo, Pooja Rao, Bruno Schmitt, Sam Stanwyck, Norm M. Tubman, Hanrui Wang, Timothy Costa

**Affiliations:** 1 NVIDIA Corporation, Santa Clara, CA USA; 2 Department of Engineering Science, University of Oxford, Oxford, United Kingdom; 3 Department of Chemistry, University of Toronto, Toronto, ON Canada; 4 Vector Institute for Artificial Intelligence, Toronto, ON Canada; 5 Department of Computer Science, University of Toronto, Toronto, ON Canada; 6 Department of Materials Science and Engineering, University of Toronto, Toronto, ON Canada; 7 Department of Chemical Engineering and Applied Science, University of Toronto, Toronto, ON Canada; 8 Quantum Motion, London, United Kingdom; 9 Department of Materials, University of Oxford, Oxford, United Kingdom; 10 Department of Physics and Astronomy, University of Waterloo, Waterloo, ON Canada; 11 Perimeter Institute for Theoretical Physics, Waterloo, ON Canada; 12 Qubit Pharmaceuticals, Paris, France; 13 Quandela, Massy, France; 14 NASA Ames Research Center, California, USA; 15 Computer Science Department, University of California Los Angeles, Los Angeles, CA USA

**Keywords:** Quantum information, Quantum simulation

## Abstract

Artificial intelligence (AI) advancements over the past few years have had an unprecedented and revolutionary impact across everyday application areas. Its significance also extends to technical challenges within science and engineering, including the nascent field of quantum computing (QC). The counterintuitive nature and high-dimensional mathematics of QC make it a prime candidate for AI’s data-driven learning capabilities, and in fact, many of QC’s biggest scaling challenges may ultimately rest on developments in AI. However, bringing leading techniques from AI to QC requires drawing on disparate expertise from arguably two of the most advanced and esoteric areas of computer science. Here we aim to encourage this cross-pollination by reviewing how state-of-the-art AI techniques are already advancing challenges across the hardware and software stack needed to develop useful QC - from device design to applications. We then close by examining its future opportunities and obstacles in this space.

## Abstract (web summary)

Quantum computing devices of increasing complexity are becoming more and more reliant on automatised tools for design, optimization and operation. In this Review, the authors discuss recent developments in “AI for quantum", from hardware design and control, to circuit compiling, quantum error correction and postprocessing, and discuss future potential of quantum accelerated supercomputing, where AI, HPC, and quantum technologies converge.

## Introduction

Quantum computing (QC) has the potential to impact every domain of science and industry, but it has become increasingly clear that delivering on this promise rests on tightly integrating fault-tolerant quantum hardware with accelerated supercomputers to build accelerated quantum supercomputers. Such large-scale quantum supercomputers form a heterogeneous architecture with the ability to solve certain otherwise intractable problems. Many of these problems, such as chemical simulation or optimization, are projected to have significant scientific, economic and societal impact^1^.

However, transitioning hardware from noisy intermediate-scale quantum (NISQ) devices to fault-tolerant quantum computing (FTQC) faces a number of challenges. Though recent quantum error correction (QEC) demonstrations have been performed^2,3^, all popular qubit modalities suffer from hardware noise, preventing the below-threshold operation needed to perform fault-tolerant computations. But even qubits performing below threshold face scaling obstacles. FTQC is demanding and necessitates more resourceful QEC codes, faster decoder algorithms, and carefully designed qubit architectures. Both QC hardware research and current quantum algorithms also require further development with explorations of more resource-efficient techniques, having the potential to dramatically shorten the roadmap to useful quantum applications.

Though high-performance computing (HPC)^4–6^, and in particular, accelerated GPU computing^7,8^, already drives QC research through circuit and hardware simulations, the rise of generative artificial intelligence (AI) paradigms^9^ has only just begun. Foundational AI models^10^, characterized by their broad training data and ability to adapt to a wide array of applications, are emerging as an extremely effective way to leverage accelerated computing for QC. While the architecture landscape of these models is diverse, transformer models^11^ have proven particularly powerful, and especially popularized by OpenAI’s generative pre-trained transformer (GPT) models^12,13^. There is already a strong precedent for these models being applied to technical yet pragmatic tasks in other fields, ranging from biomedical engineering^14^ to materials science^15^. Bringing the deep utility and broad applicability of such models to bear on the problems facing QC is a key goal of this review.

There is ample intuition to motivate exploring AI as a breakthrough tool for QC. The inherent nonlinear complexity of quantum mechanical systems^16^ makes them well-suited to the high-dimensional pattern recognition capabilities and inherent scalability of existing and emerging AI techniques^17^. Many AI for quantum applications are being realized for near-term development of quantum computers and the long-term operation of scalable FTQC workflows^18^. This review examines applications of state-of-the-art AI techniques that are advancing QC with the goal of fostering greater synergy between the two fields.

Despite the considerable promise of AI, it is critical to recognize its limitations when applied to QC. AI, as a fundamentally classical paradigm, cannot efficiently simulate quantum systems in the general case due to exponential scaling constraints imposed by the laws of quantum mechanics. Classical simulation of quantum circuits suffers from exponential growth in computational cost and memory consumption. This exponential scaling fundamentally limits the size of quantum systems that classical AI can simulate, impacting their generalizability to larger problems. For example, the GroverGPT-2^19^, which uses large language models (LLMs) to simulate Grover’s algorithm, encounters these constraints. GroverGPT-2’s ability to simulate full circuits is limited by the maximum context length of the LLM, making larger circuits infeasible. It faces limitations in generalization, with performance deteriorating for problem sizes significantly beyond the training data. This suggests that classical resource bottlenecks are effectively relocated rather than removed, contributing to scaling costs and deployment hurdles. AI in the context of simulating large-scale quantum systems serves as a complementary tool for interpreting, approximating, and reasoning about quantum processes, rather than a direct substitute for quantum hardware.

It is worth clarifying that this review focuses solely on the impact of AI techniques for developing and operating useful quantum computers (AI for quantum) and does not touch upon the longer-term and more speculative prospect of quantum computers one day enhancing AI techniques (often referred to as quantum for AI), which are surveyed in ref. ^20^.

**Fig. 1:**
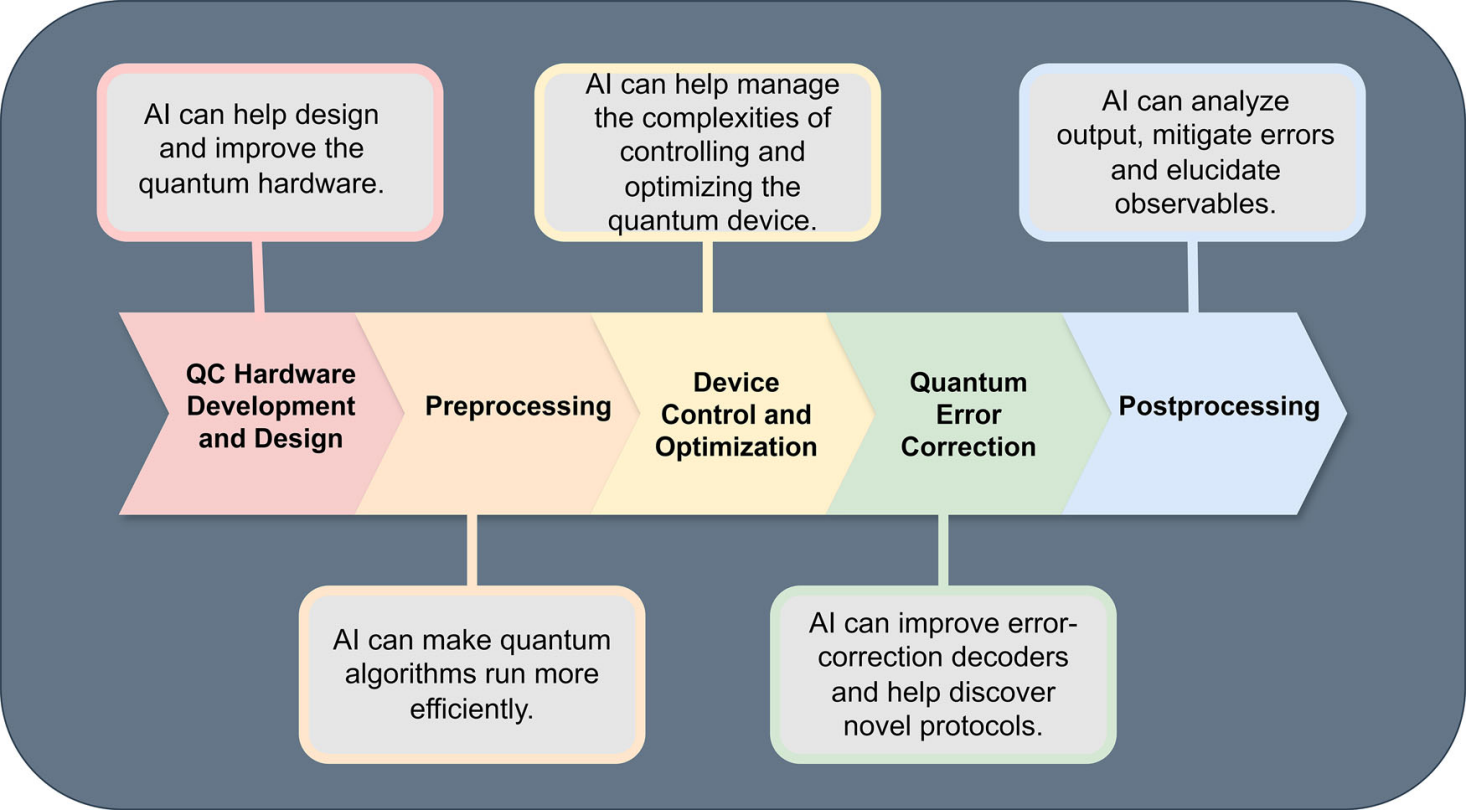
A depiction of the sections covered in this review and how AI can be used to benefit the entire QC stack. Depiction of this review's structure aligned with a generic quantum computing workflow. A brief summary of the role of AI at each stage is provided.

The content of this review is organized according to the causal sequence of tasks undertaken in operating a quantum computer (Fig. 1). We immediately stretch this taxonomy by beginning in “AI for quantum computer development and design” with how AI techniques can accelerate fundamental research into designing and improving the quantum hardware needed to operate a useful device. Then, the sections entitled “AI for preprocessing”, “AI for device control and optimization”, “AI for quantum error correction”, and “AI for postprocessing” step through AI’s roles in the widely accepted QC workflow: preprocessing, tuning, control and optimization, QEC, and postprocessing. AI’s use in algorithm development, that is, AI’s impact on various algorithmic subroutines, is covered throughout the whole manuscript, where relevant, and spans many tasks across the workflow. Each section also concludes with a discussion of the key limitations and challenges for applying AI to such use cases. The review concludes with an “Outlook” looking ahead to fruitful areas where AI might still be applied and speculating on areas of development that will further AI’s ability to solve QC’s remaining challenges.

### A brief survey of AI methods

**Fig. 2:**
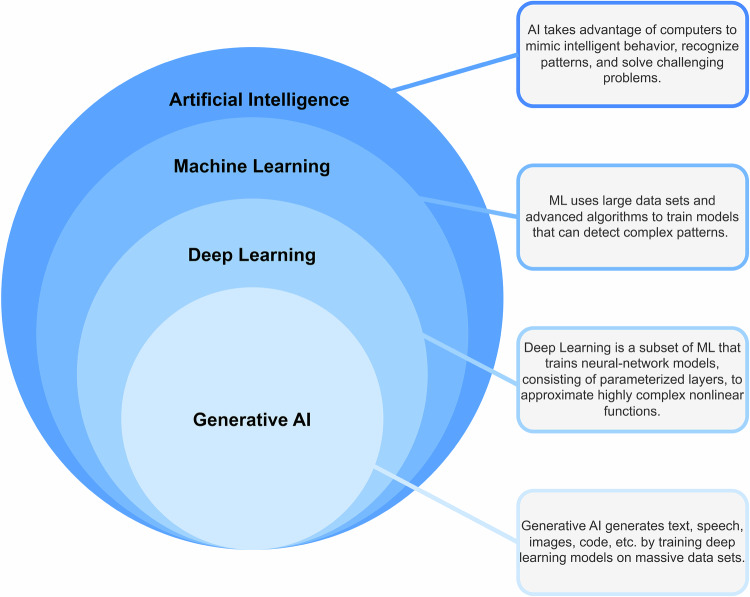
A simple hierarchy from Artificial Intelligence to generative AI, broadly contextualizing the techniques discussed in this work. Each level is paired with a simple description.

The majority of modern AI methods pertain to the subfield of machine learning (ML) (AI is a strict superset of ML, since there are AI algorithms based on hard-coded rules, which there is no training procedure that algorithmically ’learns’ from data.), consisting of algorithms that extract and utilize information from datasets^21^. Though there are many different ML architectures (decision trees, support vector machines, clustering models, etc.), in this review, we focus primarily on architectures pertaining to the field of “deep learning”, meaning that they are based on some form of deep neural network (DNN)^22,23^ which have driven recent advancements in AI. Maturing to industrial scales in the 2010’s, DNNs learn multiple data abstractions via the process of backpropagation. These data abstractions are used to construct useful representations of the dataset of interest. DNNs are characterized by their flexibility in representing patterns in data and the adaptability of their architectures. This has resulted in DNNs contributing numerous architectures to the sprawling phylogeny of ML models, which have found application across disciplines. We depict these relationships in Fig. 2.

In the broadest of strokes, we can categorize DNN applications as discriminative and generative^24^. The former seeks to learn the conditional probability distribution *P*(**y**∣**x**) of value vector **y** given feature vector **x**, whereas the latter seeks the joint probability distribution *P*(**x**, **y**). Less formally, discriminative models learn to distinguish between data types while generative models learn to produce new instances of their target data classes.

DNN architectures are incredibly diverse and ever expanding, and we here overview but a handful of them that will prove germane in this review. Among the most popular of such architectures is Reinforcement Learning (RL), which focuses on sequential decision-making^25,26^. In RL, the DNN or “agent” is tasked with navigating a learning problem and trained by assigning it a score after each decision it makes, rewarding it for useful decisions and punishing it for problematic ones. In maximizing this so-called cumulative “reward”, the agent learns how to evaluate outcomes (value) and respond to situations (policy). RL models find particular utility for automating multi-faceted tasks in dynamic environments with delayed rewards. However, RL is often difficult to implement due to its sensitivity in selecting hyperparameters.

ML tasks often focus on learning from and producing new sequences. A canonical example is natural language processing, wherein the ML agent learns from existing sentences (word sequences) to produce new ones^27,28^. For many years, such problems were addressed with recurrent neural networks (RNNs), which apply a single set of weights along the elements of a given input sequence, producing an output sequence step-by-step^29,30^. More recently, Transformer models^11,31^, including the famed GPT family^12^, have dominated sequence learning, bolstered by their parallelizability, long-range and bi-directional token (word) context, and their adroitness with variable-length inputs.

Another popular model for generative tasks is the diffusion model^32^, such as more recent DALL-E models^33^. These models use random walks with drift, as formalized by, e.g., Markov chains, to gradually add noise to target data and then learn the reverse denoising process. After such training is complete, the diffusion models can generate desired samples from noise.

Critical for training all of these deep learning methods is high-quality data. In the case of QC, this data must often be obtained via simulation with supercomputers due to noise and scale limitations of quantum computers, as well as the cost (time and economic) of obtaining quantum data. Section 15 discusses simulation in greater detail.

## AI for quantum computer development and design

Fundamental improvements in quantum hardware require a systematic engineering process that relies on precise, costly, and extremely difficult experimentation throughout the development cycle. The first step in this cycle is to design a physical system with controllable quantum behavior that can be employed as a computing device. In this section, we review the AI methods employed for designing quantum devices and the ML techniques for learning devices for future designs.

### Device design

The design of the quantum processor system requires not only physically characterizing the physical system at the materials level, but also analyzing the individual components they’re used in, which can vary widely due to inevitable irregularities (e.g., in fabrication^34^), nonidealities (e.g., crystal strain^35^), inherent material complexities^36^, and the limitations of optical components^37^. AI can accelerate the initial design and fabrication phases of quantum devices by offering insights into complex physical quantum systems.

AI approaches have been employed to successfully design multi-qubit operations by exploring potential superconducting designs that were then demonstrated experimentally^38,39^. AI can also be leveraged to generate or optimize the physical geometries of qubit circuits at the device level in solid-state devices, where learning the features in a qubit circuit can be used to generate new more exotic circuits beyond transmons, fluxonium or zero-pi qubits.^40,41^ In addition, AI can be employed to design quantum optical setups that can then be employed to generate highly entangled states^42–45^. AI models learning how to optimize the performance of multi-qubit gates in nonuniform semiconductor-based qubits can automate the handling of manufacturing variabilities in these devices^46^.

A distinct yet related challenge is designing AI methods which are dependent on information that cannot be directly included in model training, such as unavoidable classical noise or inherent quantum uncertainty^47^. Such limitations have also been addressed by iterative applications of transfer learning^48^, e.g., by pre-optimizing system control in progressively more realistic and challenging substrates^49^. The reverse tactic has likewise proven fruitful, where AI models versed in well-understood quantum systems are used to propose novel quantum experiments. This same technique can be applied to leverage AI models trained on a quantum system to extrapolate new architectures and quantum information protocols^50^.

### Learning models of quantum systems

Central to the design of future quantum devices is our understanding of today’s smaller experimental quantum systems. These systems are usually categorized as a closed system, which is driven by a structured Hamiltonian, or an open system with dissipative and non-Markovian dynamics.

Studies of the closed system model are led by the broad field of Hamiltonian Learning^51–53^, which seeks to identify the generating Hamiltonian of observed quantum dynamics through the use of ML methods. Such methods are quite generally applicable^54,55^ and applying these to characterize measurement-expensive and noise-prone contemporary quantum computers^56^ can be challenging. There has been success in meeting these constraints by refining models to require only tractable amounts of quantum input data^57^.

Unlike closed systems, open system models require learning the Lindblad master equation, which generally requires more parameters and is more complex to represent. One approach to learning such dynamics is to embed non-Markovian dynamics into a Markovian system^58^ and use ML to learn this embedding. Alternatively, neural networks can directly capture the process by parameterizing the Lindbladian operators^59^. Furthermore, with a given noise model, ML-assisted characterization has demonstrated its ability to discover two-level systems by learning the decay parameters in the Lindbladian equation^60^.

For both closed and open system models, the ML-assisted characterization of quantum systems can be greatly simplified by the inclusion of relevant information^53,61,62^, e.g., observable constraints, which combine physics equations to guide deep learning models.

**Fig. 3:**
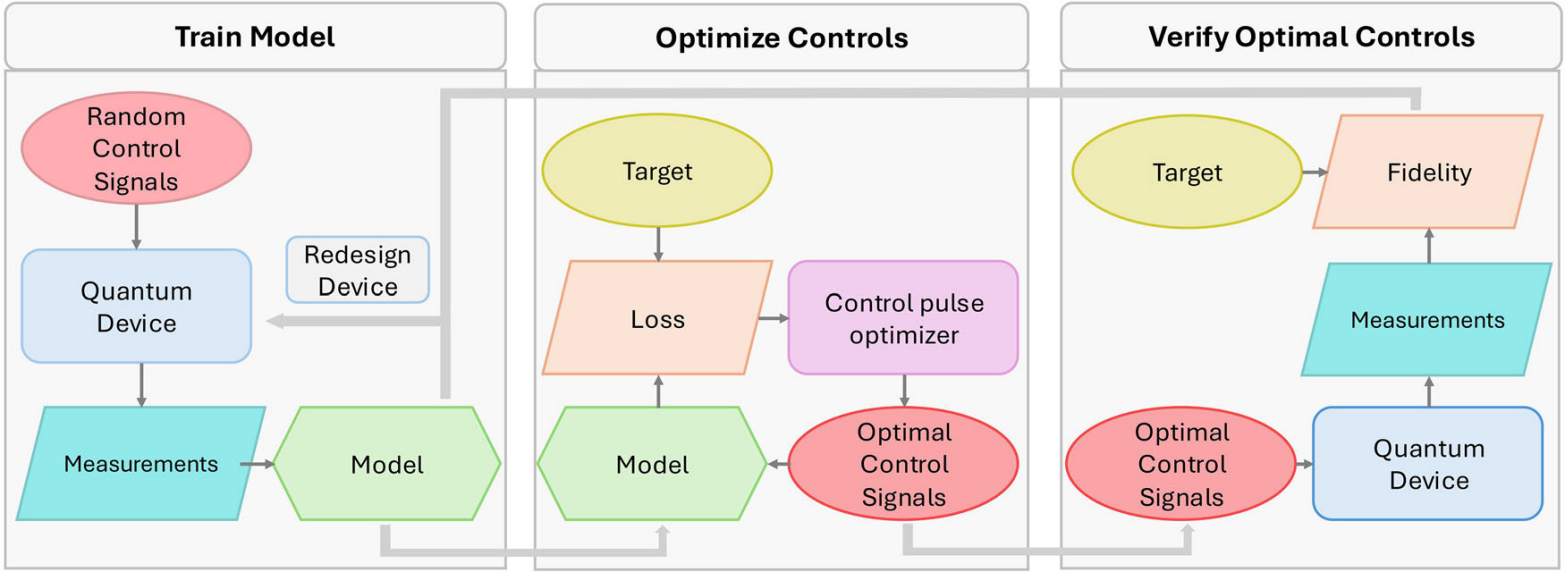
Schematic of process for training ML models, adapted from ref. ^63^. The process starts with creating an experimental or simulated dataset by applying controls to the system and recording outputs. This dataset trains ML models, which are then use to determine optimal control settings.

These physics informed ML models also have utility in more applied tasks, such as optimizing control voltages for a photonic quantum circuits (Fig. 3)^63^. More generally, ML methods have been used to learn quantum device characteristics otherwise inaccessible to experiments - such as disorder potentials^64,65^ and the nuclear environment of a qubit^66^.

### Key limitations

The main challenge for AI in quantum device design is ensuring its models accurately reflect real-world physical devices. Since the optimization of device and entangling gate parameters relies on numerical models, there is risk that these models deviate from actual hardware behavior. This issue is compounded by the scarcity of real-world datasets and the high cost and time commitment associated with generating new data. In particular, the predictive power of these AI tools is fundamentally constrained by the accuracy of the hardware approximations.

## AI for preprocessing

Preparing quantum algorithms to run on a quantum device is a significant challenge. Practical implementation of algorithms requires generating compact circuits that run as fast and efficiently as possible, whilst accounting for device-specific constraints. We refer to this process as “preprocessing". Recent advancements in AI methods have opened new possibilities for more efficient and flexible quantum circuit design. This section surveys how AI is being used to improve key preprocessing steps such as unitary synthesis, circuit reduction, and state preparation - unlocking more efficient quantum algorithms.

### Quantum circuit compilation

Circuit compilation is the orchestration of potentially hardware-specific operations to efficiently realize some desired quantum circuit. Optimizing circuit compilation is a challenging task that has specific bottlenecks due to the nature of optimizing on quantum hardware, and its complexity quickly becomes unmanageable for larger circuits. Quantum circuit compilation typically consists of four stages: circuit synthesis, where a high-level algorithm is translated into a logical gate-level circuit, circuit optimization, which reduces resource costs such as gate count or depth, mapping and routing, which adapt the circuit to hardware constraints, and gate decomposition, where gates are expressed in the hardware’s native gate set. In this section, we review several approaches that leverage AI to automate circuit compilation and overcome its challenges^67–71^.

#### Unitary synthesis

Unitary synthesis is a particularly important circuit synthesis task that prepares a quantum circuit to implement a specific unitary operation. The primary challenge is decomposing the unitary matrix representing the operation into a sequence of elementary quantum gates, typically from a universal gate set^72^. The complexity of unitary synthesis increases exponentially with the number of qubits, making exact synthesis computationally prohibitive for large quantum systems. Managing this high dimensionality alongside hardware constraints (such as variable gate fidelities and qubit connectivity) necessitates the use of approximation techniques or heuristic methods.

In recent years, AI-based methods have emerged as powerful tools for unitary synthesis. Deep learning techniques can automate navigating the vast space of potential gate sequences during the decomposition process^73^. For example, RL can treat synthesis as a sequential decision-making problem, where an agent iteratively selects gates to construct a circuit that closely approximates the desired unitary operation. There are also approaches that use multi-layer NNs to select circuit templates and propose initial parameters, achieving unitary synthesis for up to three qubits^74^.

Moreover, diffusion models^32^ - generally considered to be the precursors to transformers, have also recently been applied to generate valid circuits for arbitrary unitary operations^75^. A circuit representation is tokenized such that it can be embedded as a 3-D tensor. A conditioning step follows, which ensures that a text prompt is embedded via a pre-trained language model. This along with the unitary representation of the circuit, is fed into a convolutional neural network (CNN) architecture known as a U-Net^76^, which follows the typical diffusion model training procedure. For a given epoch, a timestep, *t*, is sampled, and Gaussian noise added to the input data based on *t*. The job of the NN is to learn the added noise via backpropagation. The trained model can then be used during inference to generate valid data distributions from noisy samples. Results in ref. ^75^ demonstrate applications to 3 qubit models with a gate set comprising of 12 gates and recent results in ref. ^77^ demonstrate up to 5 qubit circuit synthesis with continuous parameters using a multimodal diffusion model.

#### AI for circuit optimization

AI-based methods have demonstrated significant potential in improving the optimization of quantum circuits^78,79^. Google DeepMind’s RL-based approach to circuit generation, AlphaTensor-Quantum, is a noteworthy example^78^. The approach optimizes circuits by minimizing the count of notoriously expensive non-Clifford T-gates - translating the optimization problem into a tensor decomposition. To mitigate challenges associated with RL, such as exploring a large combinatorial space, training instability, and computational overhead, domain-specific knowledge is utilized, including alternative implementations of T-gates with Toffoli and controlled-S gadgets.

In the NISQ era, circuit optimization remains essential, particularly for mitigating the noise of multi-qubit gates. Since gate sets and qubit connectivity vary across hardware platforms, it is important to tailor circuits accordingly. Deep RL can autonomously discover optimization strategies that reduce both circuit depth and gate count under hardware-specific constraints^79^. Compilation pass flow has also been optimized with Markov decision process and deep learning^80^. The challenges of a large action space and non-uniform circuit representation have also been addressed by introducing a hierarchical action space and leveraging a graph neural network (GNN), a specialized NN for processing data in graph form, to represent circuits^81^. A hybrid deep reinforcement learning framework has also been proposed for compiling trapped-ion quantum circuits, where a deep learning agent selects discrete gate operations, and continuous gate parameters are optimized separately using gradient-based methods^82^. This two-level optimization enables efficient and architecture-aware circuit compilation. Compilation and quantum circuit synthesis have also been combined with an approach that uses a single RL framework, enabling scalable compilation across various circuit classes^83^.

### AI models to generate compact circuits

An important requirement for preprocessing is to generate *compact* quantum circuits. Compared to previously considered ‘brute-force’ approaches to quantum circuit generation^84^, generative AI models have demonstrated promise in generating more compact circuits^85^.

**Fig. 4:**
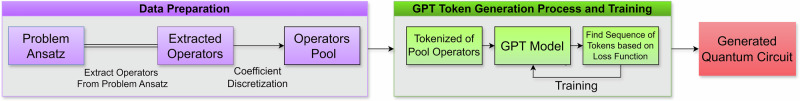
Workflow of the GPT-QE algorithm. During the initial **Operator Preparation** stage, operators are extracted (the choice of operators depends on the problem ansatz - unitary coupled cluster singles and doubled (UCCSD) and quantum approximate optimization algorithm (QAOA) being two examples), resulting in Hermitian operators ${\{{P}_{j}\}}_{j}$ such as Pauli strings. In addition, a range of discrete coefficients (${\{{\theta }_{k}\}}_{k}$) are generated. {*P*_*j*_} and {*θ*_*k*_} are combined into different unitary pool operators (${\{{e}^{i{P}_{j}{\theta }_{k}}\}}_{j,k}$). During the next **GPT token-generation and training** stage, the $\{{e}^{i{P}_{j}{\theta }_{k}}\}$ are tokenized and passed to a transformer for training. In training, the model produces sequences of tokens for which the loss function is computed. These losses are used to update the transformer parameters. Finally, after training, the model is able to generate a `prediction' of a quantum circuit.

Recent work introduced a generative pre-trained transformer based quantum eigensolver (GPT-QE) depicted in Fig. 4^85^. Here, a (GPT) model is employed to sample a quantum circuit sequence from a pre-defined pool of operators, such as unitary coupled cluster with single and double excitations (UCCSD). The transformer model parameters and energies computed from the sampled quantum circuits are used to compute a loss function. Model parameters are then updated by back-propagation. Repeating this process trains the transformer model to generate quantum circuits, minimizing the loss function. Trained GPT-QE models can be used as warm starts for adjacent problems in the same domain or to generate datasets for training other GPT models to generate novel quantum circuits for a domain-specific problem.

GPT-QE has been extended to combinatorial optimization problems through Generative quantum combinatorial optimization (GQCO)^86^ and QAOA-GPT^87^. GQCO^86^ employs an encoder-decoder transformer to generate quantum ansätze tailored to specific problem instances. Trained on multiple instances, it learns to map classical optimization problems to quantum circuits. QAOA-GPT^87^, on the other hand, uses a GPT-style model to generate compact quantum approximate optimization algorithm (QAOA) circuits after training on a synthetic dataset derived from adaptive QAOA with the same goal of producing circuits that work out-of-the-box.

### Circuit parameter learning and parameter transfer

Another important strategy that can be considered during the preprocessing stage of quantum computation is whether parameters can be *transferred* between quantum circuits. This is particularly relevant for circuits implementing the Variational Quantum Eigensolver (VQE), QAOA and other variational quantum algorithms^88–91^. Parameter transfer is the process of using optimal circuit parameters from other use cases to accelerate the generation of optimal parameters in a new, distinct use case.

Graph embedding techniques, such as Graph2Vec^92^, GNNs^93^ and GL2Vec^94^, have been used to facilitate such transferability by identifying structural similarities between graphs representing different problem instances. These embeddings allow AI models to predict optimal parameters for new instances by leveraging pre-optimized donor parameters, significantly reducing the computational overhead compared to running the optimization from scratch^95^.

This methodology is especially effective in mitigating the barren plateau problem^96^, wherein a very flat optimization landscape causes gradient-based optimization methods to struggle to navigate toward global optima - a critical issue in the training of quantum circuits. Transferability pipelines built on graph embeddings also allow the scaling of QAOA performance, with an order of magnitude improvement in efficiency under both ideal and noisy conditions^97^.

While parameter transfer between algorithms is actively studied, and it may work in certain cases, transferring circuit designs across hardware platforms is a much harder problem. Differences in gate sets, connectivity, and noise make direct reuse of circuits often impractical. Only limited work exists on hardware-aware adaptation, and systematic methods for cross-platform design transfer remain an open problem.

It is important to mention one particular set of AI algorithms used to improve ML models by using meta-learning. Meta-learning is a class of algorithms that ‘learn to learn’. These algorithms have been successfully applied for circuit initialization^98^ and circuit optimization^99^.

### State preparation

A preprocessing task prescribed by a number of algorithms is the preparation of particular quantum states. However, naïve implementations of such state preparations generally require circuits having a depth that grows exponentially with problem size^100^. This quickly becomes intractable for large-scale algorithms, motivating a more innovative approach to state preparation. This has led to the exploration of ML-assisted methods^101^ - including both classical and quantum NNs^102,103^ and other related techniques.

AI-based approaches to state preparation are broad, accommodating the many specialized heuristics, optimizations and initializations that can apply to the wide range of possible state preparation problems^104^. Many of the techniques already described in this review, such as GPT-QE and meta-learning, have been co-opted for state preparation purposes. We note that pre-optimization ideas have also been referred to as “warm-starting" and “no-optimization" with the main idea being to use heuristic or classical simulations before starting any optimization on quantum hardware^105–107^, which draw on a wide range of AI methods. As quantum algorithms become increasingly refined, optimization tasks are likely to be moved from quantum to classical hardware wherever possible - increasing the relevance of improved AI techniques for state preparation.

Particular attention has been paid to using RL for state preparation, which has proven particularly successful when employing discrete action spaces^108^. RL has been used for state preparation on both ideal^109,110^ and experimental^111^ systems, and has been used to optimize experimental figures of merit such as fidelity, gate cost and runtime. Remarkably, these approaches have also yielded theoretical insights in addition to blackbox functionality, generalizing to learn entire state classes rather than single instances^112^ and adhering to spin glass-like hardness guarantees^73^.

Finally, we highlight other optimization techniques that have been developed to search over circuit space beyond the techniques described previously in this section. While there are numerous approaches that have been considered, some of the most promising techniques include basin hopping optimization^113^, genetic algorithms^114,115^ and Bayesian optimization^116,117^. In Table 1, we summarize different state preparation approaches discussed in this section.

**Table 1:** Circuit synthesis techniques for State Preparation

Reference	Method	Related Refs.
^272^ Grimsley et al. (2019)	Search(operator pool)	
^113^ Burton et al. (2023)	Search(Basin Hopping)	
^117^ Duffield et al. (2023)	Bayesian Optimization	^116^
^115^ Chivilikhin et al. (2020)	Genetic Algorithm	^114^
^85^ Nakaji et al. (2024)	Generative AI	
^110^ Ostaszewski et al. (2021)	Reinforcement Learning	^109^
^99^ Wilson et al. (2021)	Meta Learning	^98^

### Key limitations

Although AI for preprocessing has been shown to be successful, it has encountered a variety of challenges and limitations. One of the main challenges is the scalability to large systems. For instance, the diffusion model approaches, especially those based on the U-Net architecture, require significant computational resources. This makes training and inference expensive, particularly when scaling to large quantum systems or high-dimensional unitary matrices. Furthermore, training these models often involves simulating quantum circuits classically, which is exponentially hard. This limits the scalability and practicality of training on large or highly entangled quantum systems. Another example is the GNN. In GNNs, as the number of qubits increases, the graph representation and GNN model complexity grow rapidly. This can lead to memory bottlenecks and increased training time, making it difficult to scale to large quantum systems. GNNs require a large and diverse set of training examples to learn effective parameter mappings. Generating such datasets is computationally expensive, especially when simulating quantum systems classically. Generating a large and diverse dataset for a large problem size is also the same issue for GPT-QE and the QAOA-GPT framework.

## AI for device control and optimization

All approaches to building and operating quantum processors involve control, tuning and optimization of quantum devices. Control refers to actively modifying quantum states through inputs (e.g., microwave pulses) to perform desired operations. Tuning involves adjusting device parameters to target a specific operating regime, and optimization involves refining such parameters to maximize performance metrics like coherence times, operation speeds, and fidelity. The characterization of quantum devices requires probing their properties to inform control, tuning, and optimization decisions.

**Fig. 5:**
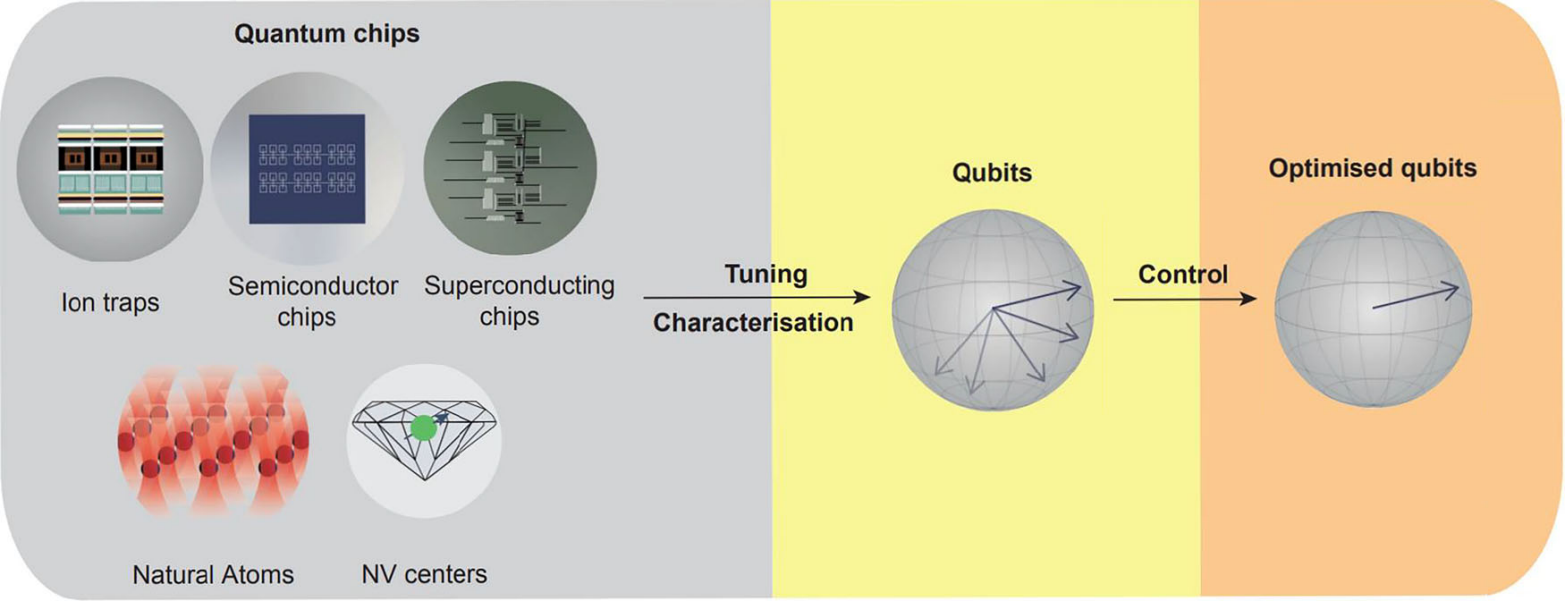
Most quantum device architectures require specific tuning and control protocols to operate as qubits. Machine learning-based approaches allow us to automate and speed up such protocols, allowing for high-throughput characterization and optimization of quantum devices.

In practice, the characterization, tuning, control and optimization of quantum devices are time-consuming processes, currently, often requiring the dedicated work of a team of quantum physicists. The use of AI approaches for automating these processes is well motivated, since NNs and Bayesian optimization methods excel at inferring appropriate outputs from limited input data without employing costly modeling from first principles. A variety of ML methods have been used to characterize different types of quantum devices, automate tuning strategies, and optimize qubit control. The ability to automate a full tuning pipeline, including the encoding of the qubit and its optimization, is a fundamental requirement for most QC platforms (see Fig. 5). Most of the automation work has focused on tackling different stages of the tuning pipeline. In Table 2, we summarize some key references in which ML-based algorithms for tuning, characterization and optimization of quantum devices have been demonstrated. In the following, we categorize the AI approaches into designing the desired quantum dynamics for implementing quantum operations, and removing the unwanted quantum dynamics to reduce the noise.

**Table 2:** Key experimental demonstrations of ML-based algorithms for quantum device tuning, characterization and optimization

Platform	Application	Approach	Reference
Semiconductor Nanowire	Fully automatic tuning of a spin qubit	BO, CNN, CV	^140^ Schuff et al. (2024)
	Qubit speed/coherence optimization	BO, CNN, CV	^141^ Carballido et al. (2025)
Semiconductor QDs	DQD tuning	DCNN	^130^ Kalantre et al. (2019)
	Qubit regime tuning	BO	^273^ Teske et al. (2019)
	DQD tuning and disorder characterization	BO	^135^ Moon et al. (2020)
	Identification of transport features	Deep RL	^131^ Nguyen et al. (2021)
	Fine-tuning of transport features	UL	^132^ van Esbroeck et al. (2020)
	Cross-platform tuning	BO-RF	^46^ Saverin et al. (2024)
	Spin readout identification	NN, CNN	^134^ Schuff et al. (2023)
	All RF-DQD tuning	BO	^136^ van Strateen et al. (2022)
	DQD tuning with charge sensor compensation	BO	^138^ Hickie et al. (2023)
	Characterization of electrostatic disorder potential	BO, CNN	^64^ Craig et al. (2024)
	Hamiltonian learning and real time qubit control	BO	^144^ Berritta et al. (2024a)
	Characterization of qubit fluctuations	BO	^145^ Berritta et al. (2024b)
NV-centers	Characterization of qubit fluctuations	BO	^147^ Arshad et al. (2024)
	Hamiltonian learning and real time qubit control	BO	^146^ Scerri et al. (2020)
	Nuclear spin sensing with a spin qubit	DL-NN	^66^ Jung et al. (2021)
Superconducting qubit	Qubit characterization	NN	^142^ Wozniakowski et al. (2020)
	Fully automated tuning of transmon qubits	LLM agents	^165^ Cao et al., (2025)
	Quantum dynamics reconstruction	RNN	^149^ Flurin et al., (2020)
	Cavity state preparation	DRL	^150^ Porotti et al. (2022)
	Qubit initialization	RL-NN	^154^ Reuer et al. (2023)
	Quantum control	Model-free RL	^122^ Sivak et al. (2023)
3D superconducting cavity	Quantum error correction	Model-free RL	^127^ Sivak et al. (2023)

### Designing optimal dynamics

Designing optimal dynamics usually involves the discovery of a set of parameters that parameterize the environment Hamiltonian, which leads to the implementation of quantum gates. It is worth mentioning the Gradient Ascent Pulse Engineering (GRAPE) method^118–121^ which is widely adopted in pulse optimization. GRAPE optimizes quantum pulses by using gradient descent to refine pulse parameters, with the predefined model of the device’s quantum dynamics and utilized for calculating these gradients and evaluating performance. However, GRAPE’s accuracy is inherently limited by the fidelity of this externally determined model. In contrast, model-free RL requires no such model. It treats the quantum system as a black box, directly leveraging feedback from the quantum hardware to learn optimal policies through exploration. It has been used to demonstrate quantum control of a superconducting qubit^122–126^ and QEC in a 3D superconducting cavity^127^. Given that the speed of the GRAPE method is faster than that of the RL method, a hybrid approach was developed for superconducting qubits. This method integrates feedback to learn dynamics while retaining gradient descent for updating pulse parameters^128^.

Many ML methods have been explored to automate and optimize the operation of semiconductor quantum dot devices. A variety of classifiers and NNs have been used to tune and identify charge transitions in large parameter spaces^129–133^ and detect Pauli Spin blockade^134^ (a step often required for spin qubit initialization and readout). Automated strategies based on Bayesian optimization have proven robust for tuning quantum dot devices from scratch (i.e., tuning from a de-energized device to a double quantum dot configuration - often referred to as super coarse tuning)^135,136^. Bayesian methods have also been used for quantum device tuning across different material systems^46,137^, and for multiparameter cross-compensation^138^, recently realized also with computer vision^139^. Recently, the development of algorithms involving the interplay of Bayesian optimization, CNNs and computer vision has allowed the demonstration of the first complete tuning of a single spin qubit^140^ and the optimization of qubit Rabi speed and coherence time^141^. Bootstrapping techniques can also be employed with these models, to further reduce the amount of input data required^142^ and enable partial inference based on the findings of previous studies. Reinforcement learning (RL)^26^ techniques were applied in optimizing the control parameters. RL agents demonstrate their abilities to construct optimized control pulses for semiconductor quantum dot qubits^143^.

Some qubit control techniques rely on characterizing the environment of a qubit. Real time learning of Hamiltonian parameters, enabled by fast adaptive Bayesian estimation, has been employed for such characterizations. In turn, this has enabled the optimization of qubit control - resulting in extended coherence times both in semiconductor^144,145^ and NV-centers spin qubits^146,147^.

In superconducting quantum circuits, NNs can be used to relax the requirement of device characterization and control complexity, instead modeling the single-trajectory output of a qubit directly, with high accuracy^148,149^. In superconducting qubits, NNs with deep and RL have been used to demonstrate cavity state preparation^150^, mid-circuit measurements^151^, quantum control^152,153^, and qubit initialization^154^.

In photonics platforms, ML-based methods have been implemented for control and characterization of both single and multiparameter systems^155,156^.

### Remove unwanted dynamics

The unwanted terms in the Hamiltonian cause the coherent error of quantum gates. One of the most widely applied approaches, dynamic decoupling (DD) is a low-overhead method to suppress the error from unwanted dynamics. An AI approach was applied to improve the DD sequences and demonstrates improvements to standard DD^157,158^. In addition, AI-automated qubit initialization protocols can be used to navigate the otherwise unintuitive process of preparing fault-tolerant operations at relatively high temperatures using Markov models^159^, suppressing system temperatures with RL^160^, or maximizing the coherence of quantum state transfer with RL^161^.

### Process automation

Devices parameters, usually, must be determined sequentially by individual experiments, and implementing such calibration requires human scientists to monitor experimental results and make decisions about the next steps. Such a workflow is labor-intensive and time-consuming. Large language models (LLMs) and vision-language models (VLMs), trained on a combination of visual media and text, have demonstrated strong capabilities in understanding human instructions and reasoning in both natural language and image data, and has been applied to many scientific discovery applications^162–164^. AI agents built on LLMs and VLMs have been successfully applied to automate the device calibration process, demonstrating its performance comparable to that of human scientists^165^.

### Key limitations

AI-assisted quantum calibration and control still face significant limitations. While well-trained models perform effectively, data collection remains a major hurdle, particularly the lengthy training required for RL methods. Open-loop methods, which takes a fixed model parameterized by a few key parameters of the hardware devices, often struggle to achieve high fidelity due to inherent modeling inaccuracies. Conversely, closed-loop optimization systems, which use observables as feedback, are constrained by the considerable time needed for data acquisition from quantum hardware, leading to excessively long calibration implementation times. LLM agents for automation cannot yet fully recover from all error cases, still requiring human intervention.

## AI for quantum error correction

Scalable quantum error correction (QEC) is a critical prerequisite for FTQC, yet it is extremely difficult to realize in practice. The following sections explore how AI may improve the demanding decoders needed to run QEC and help accelerate the discovery of more efficient QEC codes^166^.

### Decoding

QEC protocols involve making joint measurements on sets of qubits (syndrome qubits) and using these results to infer which physical qubits (data qubits) have most likely experienced errors. Locating errors allows them to be corrected or otherwise accounted for in the remainder of a computation. Equivalently, syndrome measurement results can be used to infer the signs of relevant logical observables after the data qubits have been measured in some basis. The inference step of this process is performed by a classical decoding algorithm, which has the difficult task of not only making a best-guess of error locations (or signs of logical observables) from limited information, but furthermore must do so with high enough speeds to prevent an insurmountable backlog of errors^167–170^. Furthermore, the choice of a particular decoding algorithm plays an important part in determining the noise threshold below which a QEC code can suppress errors.

Given the above, QEC decoders face serious scalability challenges. Moreover, given the variability of noise models across different hardware qubit architectures, a good decoder will be required to maintain its ability to correct many fault patterns across these architectures. The strict time frames in which decoding operations must be completed are governed by qubit coherence times and further constrained by connection latency between the decoder-running classical hardware and QPU^171^. Simulations have confirmed that as quantum systems scale, decoders struggle to meet the required low-latency thresholds^172^. This problem is exacerbated by increasing qubit count, complexity of QEC codes and fault-tolerant logic (implemented say via lattice surgery^173,174^). Furthermore, decoders often operate under the assumption of a simple noise model, typically a depolarizing channel. In practice, there are many far more complex real-world noise models, some of which include correlated errors, leakage and loss. These considerations make it challenging for practical decoders to adapt effectively without suffering from significant performance loss.

A diverse array of AI techniques are being explored as tools to improve the efficiency, accuracy, and scalability of QEC decoding algorithms^166,168,175–189^. The majority of this work targets the surface code and other topological codes with some of these decoders showing great promise for scalability. AI based decoders are also being explored for other quantum LDPC (qLDPC) codes, as shown for instance in ref. ^190^.

AI-powered decoders were pioneered in work leveraging Boltzmann machines to decode various stabilizer codes with the ability to generalize between codes^175^. Later, a CNN approach was used to decode higher-dimensional QEC codes, and showed strong evidence of scalability over other ML decoders, whilst also requiring less retraining as system sizes were varied^181^. This CNN approach assigns local likelihoods of errors, producing a threshold of around 7.1% for the 4D toric code under noiseless syndrome measurements. Subsequent work applied a CNN with binarization to realize higher efficiency^182^. More realistic approaches for scalability have been developed where the AI based decoder acts as a pre-decoder and an algorithmic decoder, such as minimum-weight perfect matching (MWPM), corrects residual errors. Such an approach leveraged 3D convolutions to include the temporal dimension to correct errors arising from a full circuit-level noise model. In addition, strategies such as syndrome collapse and vertical cleanup resulted in substantial speedup for implementing an MWPM decoding algorithm. This approach has been demonstrated on a distance 17 surface code, running the protocol on local FPGA hardware^168^.

Similarly, a long short-term memory recurrent neural network (LSTM RNN) trained on experimentally accessible data outperforms a traditional MWPM surface code decoder by capturing correlations between bit-flip and phase-flip errors^178^. This approach adapts to the physical system without requiring a noise model and maintains performance over multiple error correction cycles.

**Fig. 6:**
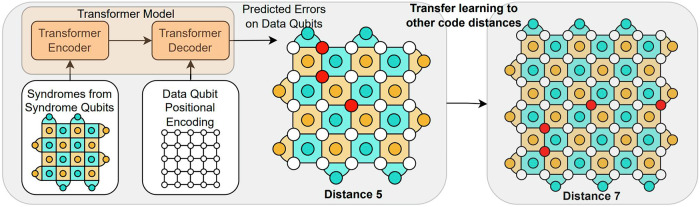
Transformer model for decoding a surface code patch. Figure adapted from ref. ^183^. Models trained on a small code distance (5 above) can transfer to larger distances (7 above) thanks to the variable input length of the transformer, cutting down on the training time.

The first application of attention-based transformer models to decoding was presented in 2023, producing a logical error rate below baselines given by MWPM and Union Find decoders^183^. As shown in Fig. 6, a transformer encoder is used to embed syndrome information and a transformer decoder is used to predict errors on each data qubit. Additional work added recurrent units to a transformer decoder and outperformed traditional approaches when trained on data from Google’s Sycamore processor for code distances 3 and 5^166^. Transformer-based decoders are currently being developed at NVIDIA for correcting errors during magic state distillation (MSD) protocols, in particular for settings such as in ref. ^191^.

Formulating decoding as a graph classification problem has also allowed GNNs to outperform traditional matching algorithms on the surface code, using simulated data and not requiring an explicit noise model^184^. This GNN approach achieved competitive performance on experimental repetition code data. Though GNN training is onerous, inference is efficient and scales linearly, indicating great promise for fast, accurate, and noise model-free decoding in practical error correction. GNN decoders have also proven successful for qLDPC codes^185^, outperforming traditional belief propagation and ordered-statistics decoding process. They have also demonstrated favorable transfer learning properties that allow decoding of high-distance codes with models trained on low-distance codes.

More sophisticated approaches like RL have also shown great utility for decoding. RL has demonstrated how an ML technique known as deep Q-learning can be applied to develop decoders that work with faulty syndrome measurements^179^. RL deep Q-networks have been used to decode toric code bit-flip errors with comparable performance to the MWPM algorithm for small error rates^180^. Besides directly performing the decoding task, other work^186^ leverages AI models to adjust the weights in the decoding graph for drifted and correlated errors.

Another more advanced approach for QEC is a recurrent-transformer-based neural network called AlphaQubit (see ref. ^166^), designed to decode surface code errors. AlphaQubit uses sophisticated noise models for initial training, followed by refinement using experimental data from Google’s Sycamore quantum processor, achieving better performance than current state-of-the-art algorithmic-based decoders.

In summary, AI-based methods for QEC decoding offers significant potential to transform the field by addressing both scalability and adaptability challenges that conventional MWPM decoders struggle with. By leveraging advanced architectures such as CNNs for spatial error likelihoods (and for correcting local error chains), transformers for syndrome embedding and state of the art performance, and GNNs for graph-based error localization-AI-powered decoders can achieve superior accuracy and faster inference without relying on different noise models. These methods adapt dynamically to complex noise environments, capturing correlations and variations more effectively than MWPM. They demonstrate strong evidence of outperforming traditional methods, providing a promising pathway toward efficient, scalable, and noise-resilient quantum error correction crucial for practical QC.

### Code discovery

The discovery of new QEC codes is crucial for advancing FTQC, especially as we strive to design codes that are more efficient, robust, and tailored to specific quantum hardware architectures. Traditionally, finding optimal QEC codes has been a labor-intensive process, relying on analytical approaches and domain expertise to explore the vast space of possible code structures. However, the complexity of quantum systems and the diversity of noise environments pose challenges that can be difficult to address with conventional methods. AI offers a promising alternative by automating the search for new QEC codes, leveraging its ability to identify patterns and optimize structures within high-dimensional spaces. ML models, such as NNs and RL agents, can explore code design spaces far beyond human intuition, identifying novel error-correction schemes and optimizing parameters to meet desired performance criteria. This data-driven approach can potentially accelerate the discovery of QEC codes that offer improved error thresholds, scalability, and adaptability to real-world noise models, thus playing a critical role in the future of quantum computation.

RL has been used for discovering various QEC codes and can be enhanced, for example, to encode circuits tailored to specific hardware^192^ in which RL agents have been shown to scale to 20 qubits and distance-5 codes. Noise-aware meta-agents can enable learning across various noise models, accelerating the discovery process. RL can also be used to optimize tensor network code geometries for discovering stabilizer codes^193^. Using the projective simulation framework, the RL agent efficiently identifies optimal codes, including those with multiple logical qubits, outperforming random search methods. For instance, this RL agent finds an optimal code 10% of the time after 1000 trials, compared to a theoretical 0.16% from a random search - giving a 65-fold performance improvement. Other work^194^ has leveraged RL in the Quantum Lego^195^ framework, focusing on maximizing code distance and minimizing logical error under biased noise. In this case, an RL agent discovers improved code constructions, including an optimal $\left[\left[17,1,3\right]\right]$ code that better protects logical information. This approach allows for tailored code design without the need for explicit noise model characterization.

### Key limitations

Despite the successes of AI-based decoders, such decoders come with their own challenges. Of particular relevance is the required training data to scale AI-based decoders to large code distances, a problem which becomes further exacerbated when performing logic such as lattice surgery, where surface code patches can grow to very large distances.

For instance, the AlphaQubit decoder of ref. ^166^ used 2 billion training examples to generate out-performance with a distance 9 surface code. To maintain out-performance over MWPM at a code distance of 11, 10^10^ training samples were required (see Fig. 3 in ref. ^166^). The authors further estimated that for a distance 25 surface code, 10^13^ − 10^14^ training examples would be required, which represents an exponential growth in training data requirements as a function of the code distance. The issue thus compounds rapidly in settings such as lattice surgery, which requires large surface code distances when measuring multi-qubit logical Pauli operators. Further, surface code patches can take a wide variety of shapes during lattice surgery operations (and contain domain walls and twists). Thus, any scalable AI-based decoder will require the flexibility to correct errors for a variety of surface code patch geometries.

NN pre-decoders, such as the ones considered in refs. ^168,188^, are able to circumvent the large training data requirement by using an architecture well adapted for correcting local error chains in both space and time. Further, such a trained network can be applied to arbitrary surface code distances. However, since such decoders can only correct local error chains of some fixed size (determined by the kernel sizes of the convolutions) and are probabilistic in nature, they need to be supplemented with an algorithmic decoder such as MWPM or Union-Find (UF)^196^. As such, to offer an appreciable speedup, the latency between the two decoders needs to be small, along with an efficient implementation of the convolutions on GPUs or FPGAs.

Fault-tolerant quantum computers capable of running practical quantum algorithms will require code distances ranging from approximately *d* = 13 to *d* = 30^174,197^. Further, such codes must allow for the fault-tolerant implementation of a universal gate set given within the limits imposed by a particular hardware architecture. Finding codes satisfying these requirements remains an important challenge when using AI for code discovery. Further work will be required to allow discovery of large-distance code with favorable properties for fault-tolerant logic.

## AI for postprocessing

Quantum applications commonly require a post-processing stage to extract meaningful results from quantum measurements and optimize the measurement process. The following sections explore how AI may improve efficient observable estimation, tomography, and readout measurements and how it can be applied to error mitigation techniques.

### Measurement state discrimination

A quantum device stores information in the physical state of a qubit. For readout, we acquire and discriminate signals from this state, mapping them to the outcomes 0 and 1. This mapping process is known as state discrimination. Depending on the quantum system, these acquired readout signals can contain rich information. This makes AI techniques well suited for enhancing the fidelity of state discrimination.

In superconducting qubit systems, readout signals are acquired as time series of probing microwave pulses. The standard approach for discriminating qubit states involves aggregating the full time series and then establishing a decision boundary based on these aggregated data. However, AI methods have improved the accuracy of state discrimination by directly analyzing these time series data. Statistical approaches such as linear discriminant analysis (LDA), quadratic discriminant analysis (QDA), and support vector machines (SVMs)^198^ have already demonstrated improved performance over the typical aggregation method. Furthermore, Hidden Markov models (HMMs)^199^ have proven effective in detecting transition events during measurement, which helps to boost readout fidelity.

Beyond statistical methods, feedforward neural networks (FFNNs), have been effective in reducing measurement crosstalk^200^ and enhancing assignment fidelity^151,201^. An improved NN model, Autoencoders^201^, further demonstrates the advancements in improving measurement fidelity.

In addition, engineering features from the readout signal can enhance the accuracy of simpler AI models. The path-signature, a stochastic time-series analysis tool, has proven effective in capturing during-measurement state transition events, thereby improving the readout fidelity of both initial and final measurement states^202^.

In neutral atom QC systems, readout signals are captured as grayscale images using high quantum efficiency image sensors. CNNs have significantly improved single-qubit measurement accuracy, reducing readout errors by up to 56% compared to traditional methods^203^.

In trapped-ion systems, readout signals are obtained by detecting fluorescence events from the ions. Neural networks have been applied to the photon count time series data to enhance state classification^204^.

These advancements in readout are especially important for FTQC as QEC relies on high-fidelity readout. Furthermore, conventional QEC often demands non-demolition measurement, where the qubit’s state must remain the same after readout to enable further operations on this qubit. AI offers a novel strategy to address this demolition error by simultaneously providing the qubit’s state at the beginning and end of the measurement^199,202^. Thus, AI helps to move closer to the precision levels necessary for FT operations.

While AI methods show promise for state discrimination, they haven’t yet been implemented for the real-time, mid-circuit readout that QEC demands. Only simple models, such as shallow FFNN, have been demonstrated in real-time feedback applications^151^. These AI models typically require longer evaluation times than simpler models, and it remains to be verified if they can meet the strict timing requirements of quantum systems.

### Efficient observable estimation and tomography

Estimating quantum observables is a key part of quantum computations, wherein quantum information is reduced into readable, classical information. Such measurement data comprises the entirety of what we may probe about a quantum system, but can be costly to obtain. Estimating an observable to some required accuracy entails combining samples from multiple measurements - with the number of required observables and samples scaling (possibly exponentially) in the system size under consideration^205^. As such, ML and AI techniques have proven useful for reducing the quantity of data points needed to estimate a given observable, using the blackbox structure of AI models for more efficient inference.

Even in the case of simple models of a single observable, such as the reconstruction of a Rabi oscillation in a spin system, ML methods have been seen to improve measurement efficiency by reconstructing state populations with smaller sample sizes^206^.

The measurement overhead of full quantum tomography^207^ is even more computationally intensive than the estimation of quantum observables. Full state tomography (FST) is, in fact, impractical for all but the smallest quantum systems. Quantum state tomography (QST) has emerged as a more feasible solution to FST by focusing on alternative approaches like shadow tomography that address the exponential scaling cost of FST^208^. One of the most promising variations of QST is the use of NN-based approaches for state reconstruction. NNs can be applied to learn quantum states from limited measurement data, making them suitable for learning the state of large systems. A good example is ShadowGPT, which uses simulated shadow tomography data to train a GPT based model for predicting Hamiltonian ground state properties^209^.

CNNs can be used for the reconstruction of high-fidelity quantum states with a fraction of the data that is traditionally required. For example, when applied to ground states of the transverse-field Ising model, a CNN-based tomography scheme achieves a tenfold reduction in observable estimation error compared to conventional maximum likelihood methods^210^. This demonstrates enhanced accuracy with only polynomial scaling resources.

NN’s can also be used in adaptive QST, through a neural adaptive quantum tomography (NAQT) method^211^. This is an adaptive framework that applies RNNs to replace computationally intensive Bayesian updates. This use of RNN’s dynamically optimizes the measurement strategy, allowing it to efficiently approximate the quantum state with far fewer resources. These efficiencies are critical for scaling QST to larger systems.

In addition to QST, one can characterize quantum operations through quantum process tomography (QPT). However, QPT typically assumes that the state preparation and measurement (SPAM) errors are negligible, which is usually not the case for practical systems. Reliable estimation requires gate-set tomography (GST). GST simultaneously estimates multiple quantum processes, the initial state, and the measurement operators, typically using maximum likelihood estimation methods with gradient-based optimizers. This process is computationally intensive and generally scales only to a few qubits. Although some practical assumptions can be made to reduce the search space^212–214^, the reconstruction remains challenging. Transformer models have been adopted to bootstrap error characterization and enable more scalable GST reconstruction in multi-qubit systems^215^.

### Error mitigation techniques

Quantum error mitigation (QEM) is a set of techniques that attempt to deal with noise in quantum systems without resorting to the full machinery of FTQC, or indeed to extend the reach of FTQC when the error rate remains finite. At any given moment in time, there is always a maximum size of the computation we can perform, limited by the achievable qubit number and error rate. QEM enables us to further increase the size of the computation with the same hardware at the cost of more circuit runs. This is especially powerful in the NISQ and early fault-tolerant era, but will continue to be useful until we build quantum computers so large that the logical qubit error rate is negligible for all possible computations of interest^216,217^. QEM spans a wide range of techniques that leverage various aspects of the computations^218–222^ such as gate noise^223–225^.

Traditional approaches like zero-noise extrapolation (ZNE) rely on sets of hyperparameters obtained by device calibration or extracted from optimizing over a set of training circuits^226,227^. Conventional ZNE constructs a model of how an observable’s expectation value varies with noise, fitting parameters to this model by probing the expectation value at different circuit noise levels. The direct application of NNs to QEM (in a similar fashion to ZNE) has also been explored^228,229^. Instead of explicitly constructing a model, the noisy expectation values of different circuit sizes can be directly input into a multi-layer perceptron, which then outputs the noiseless values of larger-size circuits. This allows one to train the NN on small classically simulable circuits, and then use it to make predictions for large, non-simulable, circuits. This approach has been further extended to other ML methods like random forest and GNNs, allowing comparison against conventional ZNE for practical problems pertaining to physical hardware with up to 100 qubits^230^. Random forest models outperform other ML techniques, including (linear) ZNE in all cases. Extrapolating random forest models to make predictions beyond circuit sizes within their training dataset leads to a distinct increase in errors. This problem has been approached by including larger training circuits with target expectation values supplied by hardware experiments obtained by applying conventional QEM^230^. In this way, the ML model can mimic the behavior of the supplied QEM techniques and lead to a lower sampling cost in experiments.

QEM can also be applied to NN tomography (see subsection ’Efficient Observable Estimation and Tomography’). Suppose one has found the circuit for preparing the ground state of some given Hamiltonian, but the output state is corrupted by noise. Approaches have been suggested^231^ for first characterizing the output noisy state using NN tomography and then further optimizing parameters in the NN to minimize the energy and mitigate the errors, in a similar spirit to subspace expansion^225^. Note this requires using an autoregressive network so that the NN states can be sampled from directly in order to perform energy optimization.

The various methods for applying AI to QEM described above have proven extremely fruitful but are still relatively limited in their scope compared to the large variety of conventional QEM techniques. However, as the field continues to mature and offers more complete noise models together with more detailed circuit structures, there is the possibility of novel future applications of AI in QEM.

### Key limitations

Even though the sampling overhead of QEM can be reduced by AI, as mentioned in “Error mitigation techniques”, the overhead will still scale exponentially with the amount of noise in the whole circuit, which is a general feature for all QEM methods. Thus, they will not be efficient for asymptotically large systems. However, for finite size systems considered in practice, QEM can still be very powerful^216,217,232^. At the moment, there is also no rigorous bound on the resultant bias in the QEM estimator constructed using AI. In particular, current AI-based methods often lack statistical guarantees beyond bias, such as variance control or confidence intervals, which limits their applicability in precision-sensitive tasks. The models are often trained on synthetic data, and if the noise model is not accurate or absent, it may result in poor, unreliable generalization on real quantum devices. This difference between training and deployment conditions can lead to unpredictable behavior and unquantified errors. But these limitations are solvable and present an important direction for further investigation.

## Outlook

The research surveyed in this work demonstrates how AI has the potential to enable breakthroughs in virtually all aspects of the development and operation of quantum computers. AI techniques are not only useful in NISQ-era devices and applications, but will also play an essential role in the building of large-scale FT machines. The exploration of how AI can be of utility for quantum computing has only just begun, and by focusing more on these techniques, the quantum community stands to see further breakthroughs in the challenges facing useful QC. In this section, we raise awareness of a number of areas of development that can catalyze improvement and further adoption of AI in QC.

### Accelerated quantum supercomputing systems

A prerequisite for researching and deploying AI models for quantum research is access to supercomputing resources. Increasingly sophisticated AI techniques require greater processing power to train, and classical computing capabilities will need to scale alongside developments in quantum hardware. This has driven international efforts to prioritize quantum research and in many cases, integrate physical quantum hardware within AI supercomputing infrastructures^233,234^.

Such integration of quantum processors within AI supercomputers is widely accepted to be a necessary architecture for building large-scale, useful quantum computers. But doing so requires specialized programming models^235^ and in some cases, additional specialized hardware^172,236^. Applying classical supercomputing to scaling challenges facing QC, such as QEC, requires extremely low-latency interconnects between collocated classical and quantum hardware.

**Fig. 7:**
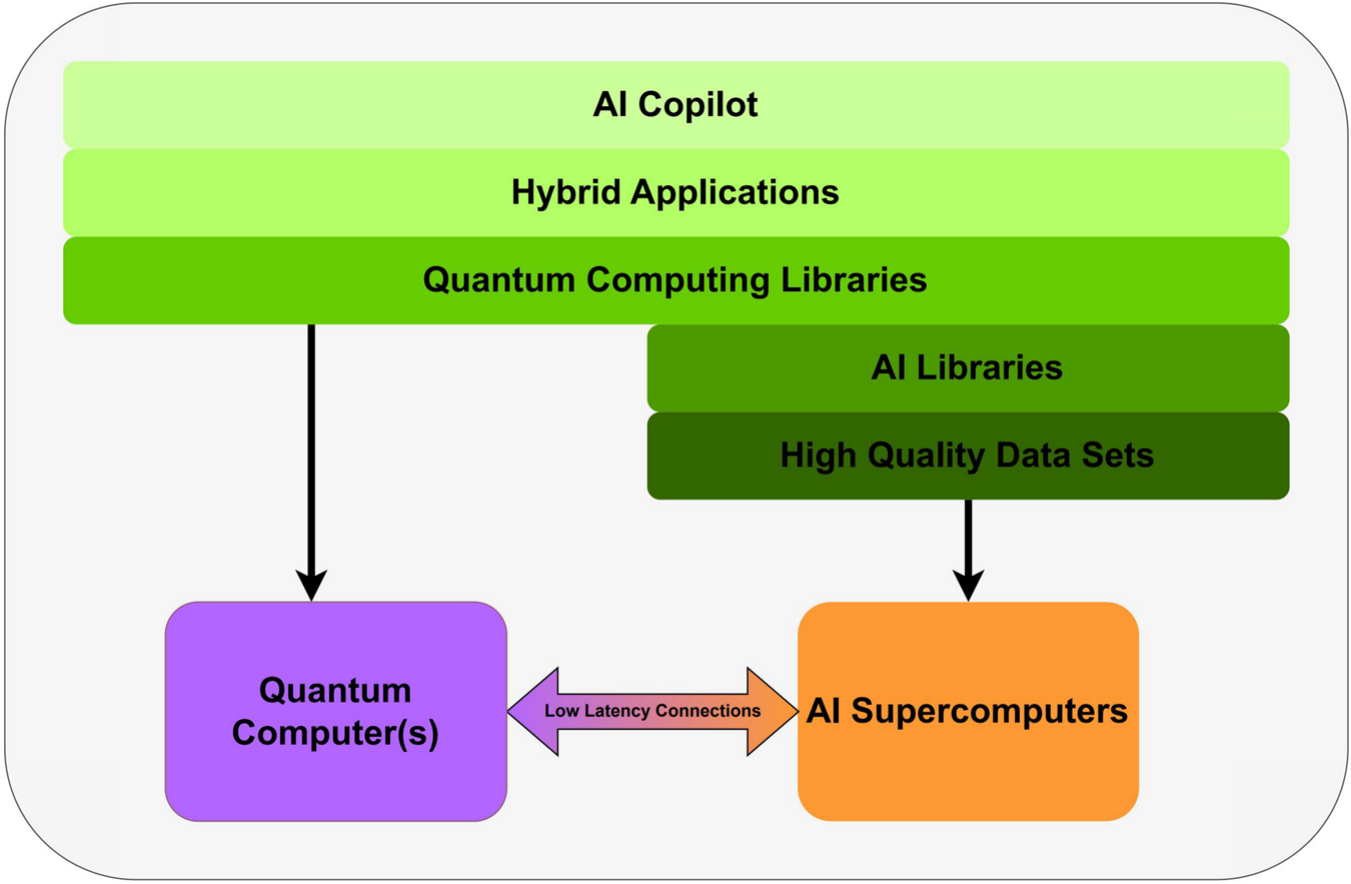
Depiction of a development platform incorporating access to both QC and AI resources. Such a platform should be accessible to both domain scientists and quantum developers, and must orchestrate hybrid workflows leveraging both AI supercomputers and quantum processors.

Development platforms for quantum-classical architectures must provide user-friendly hybrid programming workflows able to maximize performance across a heterogeneous compute architecture (see Fig. 7). To ensure adoption, such a platform should support popular scientific computing and AI libraries as well as those specialized libraries required for domain-specific applications and quantum devices control concerns. Given the broad and interdisciplinary set of users for such a platform, AI copilots will likely play a particularly important role in reducing the barrier to entry for domain experts unfamiliar with quantum application development^237–239^. In addition, AI agents could automate entire workflows^165^ much like recent tools in quantum chemistry^163^, and quantum device calibrations^165^.

### Simulating high quality data sets

Many applications of AI models for quantum computing must be trained on large, high-quality data sets. This often entails experimental data from quantum systems, which is costly to obtain given the limited availability and capabilities of current quantum processors. There are efforts to democratize and reuse existing quantum data^240–242^. But though this encourages collaboration and transparency, such efforts are unfortunately unlikely to keep up with the increasing demand for training data.

This shortfall can be addressed by synthetic training data, obtained through simulation. Simulating quantum systems can come at exponential computational cost, but is a necessity while quantum hardware is still limited in scale, quality, and availability. Conventional supercomputing resources can be used to simulate quantum processors through techniques such as quantum dynamics, digital density matrix, quantum trajectories (SV and TN)^243–248^, and large-scale (Clifford, stabilizer, Pauli paths)^249–253^. GPU-acceleration of device toy models can also be used to generate large training datasets with hundreds of thousands of samples^254,255^. As listed, these techniques can simulate systems of increasing scale, though at the cost of accuracy. Powered by accelerated computing, they can generate large and diverse data sets necessary capable of fueling the applications covered in this review^256^.

Simulation heuristics can improve the quality of synthetic data generated by simulations, and the efficiency with which it is created. ADAPT (Automatic Differentiation Adapted Quantum) methods, for example, iteratively construct quantum circuits based on the gradient of a cost function with respect to the gate chosen from an operator pool, which tends to provide resource-efficient circuits, which can be used to train other models. The methods mentioned in “AI models to generate compact circuits” provide similar benefits.

AI can also be used to simulate quantum systems directly and has driven substantial scientific progress not only in the field of QC, but in condensed matter, quantum chemistry, and similar fields. Neural quantum states (NQS) are among the most widespread of such simulation strategies^257–260^. NQS are large classical NN’s that can be sampled like a quantum system to generate data. Unlike data-driven applications, however, NQS can also be optimized *variationally*, with knowledge of a quantum system’s Hamiltonian or Lindbladian. For many applications, NQS have the potential to provide a more compact and scalable representation than other many-body techniques, such as tensor networks, but the benefits are still under investigation^261^.

Several additional AI models have been developed specifically to solve time-dependent quantum dynamics. For example, the Heisenberg equations of motion can be modeled via deep NNs^262,263^. More recently, Fourier Neural Operators (FNOs)^264^ have been deployed to learn the time-evolution of quantum spin systems. Of particular interest is the ability of FNOs to extrapolate to predictions spanning longer times than are seen in training, potentially extending quantum evolution past the capacities of modern devices or tractable tensor network depths. In addition, gradient-based optimization protocols and Bayesian inference combined with differentiable master equation solvers have been proved useful to compute steady state solutions and time evolutions of open quantum systems^265^.

### Increased Multidisciplinary Collaboration

Availability of computational resources, development platforms, and quality data sets are all foundational for enabling AIs application to QC research. But another key gating factor is collaboration between AI and QC experts. It is likely that many of the most cutting-edge AI techniques have not yet had their greatest impact on developments in QC, and many areas remain where deep collaboration may yield new breakthroughs. Here, we highlight several areas in AI research we believe hold great potential for future exploration.

Diffusion models (introduced in “A brief survey of AI methods”) have proven incredibly impactful in other application areas, but have so far only been applied to unitary synthesis^32^ for quantum computing (see “Unitary synthesis”). There is also the opportunity to apply recent training methodologies to problems in the development of quantum computing. For example, Advanced RL techniques have been proposed to solve real-world problems (See refs. ^266,267^ as examples). Whilst generative flow networks^268^ combine RL and generative models in an effort to incorporate reasoning in ML-based exploration. Both of these techniques are potentially useful in QC, where RL is already being applied.

Perhaps one of the more ambitious uses of AI for quantum, and a prime opportunity for collaboration, is the design of new quantum algorithms. Quantum algorithm design is an extraordinarily difficult task, and could rightly be regarded as a grand challenge in science. All currently known quantum applications rely on a small set of algorithmic primitives, which has not been significantly expanded for many decades. One future strategy is the use of AI to generate novel quantum algorithms. Such a process could begin by defining a scientific problem and then working backward to generate the quantum circuits needed to provide a solution according to some figure of merit. Optimistically, one could hope that beyond an application-specific algorithm, such an approach might also lead researchers to entirely new algorithmic primitives and thus unlock whole new classes of quantum algorithms. This kind of AI-assisted approach to algorithm discovery would represent an evolution of the AI-driven circuit synthesis methods described in “Quantum circuit compilation” and other alternative approaches^269–271^.

It may also be the case that incorporating AI into the algorithm design process could lead to the generation of more fundamentally hybrid quantum algorithms, that draw more optimally on both quantum and AI-based algorithmic primitives. Continued collaboration between the AI and quantum computing communities could easily also result in the development of new models developed specifically for QC applications. The development of quantum-specific foundational models may see the greatest AI-enabled breakthroughs in QC yet.

## Conclusion

QC is experiencing an explosion of utility from AI. The research surveyed in this review demonstrates that AI can play a role in everything from designing qubits, preparing efficient quantum algorithms, controlling and calibrating the device, correcting errors in realtime, and interpreting the output from QC. Most importantly, each aspect of QC needs to scale, and AI might be the only tool with the ability to both solve these problems effectively and do so efficiently at scale. AI has only begun to benefit QC, and it is likely that AI will play an increasingly critical role into the realization of useful QC applications and FTQC.
